# Supplementary material for: Investigational medicinal products, related costs and hospital pharmacy services for investigator-initiated trials: A mixed-methods study
Source: PLoS One. 2022 Mar 4;17(3):e0264427. doi: 10.1371/journal.pone.0264427 (PMC8896670; doi:10.1371/journal.pone.0264427)
Supplement: S1 Appendix — (DOCX) [file pone.0264427.s001.docx]

**Investigational Medicinal Products, related Costs and Hospital Pharmacy Services for Investigator-Initiated Trials: A mixed-methods study**

**S1 Appendix. Search strategy**

*Search strategy related to IMP costs*

Database: Ovid Medline(R) ALL

1. drugs, investigational/ or placebos/ or solutions/

2. (Cost or Costs or Budget* or Expens* or Expenditur* or Money* or financ* or Payment*).ab,kw,ti.

3. 1 and 2

4. (Placebo? or Drug? or Medication? or Medicine? or Pill? or Capsule or Tablet or Pharmacy or Pharmacies or Intervention? or IMP or investigational medical products or investigational medicinal products).ab,kw,ti.

5. ((Pharmaceutical or pharmacological or medicinal or medical or investigational or therapeutic) adj2 (product? or preparation? or compound? or Powder? or solution? or device or inhaler)).ab,kw,ti.

6. 4 or 5

7. ((Placebo? or Drug? or Medication? or Medicine? or Pill? or Capsule or Tablet or Pharmacy or Pharmacies or Intervention? or IMP or investigational medical products or investigational medicinal products or ((Pharmaceutical or pharmacological or medicinal or medical or investigational or therapeutic) adj2 (product? or preparation? or compound? or Powder? or solution? or device or inhaler))) adj4 (Cost or Costs or Budget* or Expens* or Expenditur* or Money* or financ* or Payment*)).ab,kw,ti.

8. "Costs and Cost Analysis"/

9. 6 and 8

10. 3 or 7 or 9

11. Controlled Clinical Trials as Topic/ or clinical studies as topic/ or Randomized Controlled Trials as Topic/ or clinical trials as topic/ or clinical trial? as Topic.mp. or randomised controlled trial? as Topic.mp. or research design/mt, st or Biomedical Research/mt, st

12. 10 and 11

13. limit 12 to yr="2015 -Current"

Database: Ovid Embase

1. drugs, investigational/ or placebos/ or solutions/

2. (Cost or Costs or Budget* or Expens* or Expenditur* or Money* or financ* or Payment*).ab,kw,ti.

3. 1 and 2

4. (Placebo? or Drug? or Medication? or Medicine? or Pill? or Capsule or Tablet or Pharmacy or Pharmacies or Intervention? or IMP or investigational medical products or investigational medicinal products).ab,kw,ti.

5. ((Pharmaceutical or pharmacological or medicinal or medical or investigational or therapeutic) adj2 (product? or preparation? or compound? or Powder? or solution? or device or inhaler)).ab,kw,ti.

6. 4 or 5

7. ((Placebo? or Drug? or Medication? or Medicine? or Pill? or Capsule or Tablet or Pharmacy or Pharmacies or Intervention? or IMP or investigational medical products or investigational medicinal products or ((Pharmaceutical or pharmacological or medicinal or medical or investigational or therapeutic) adj2 (product? or preparation? or compound? or Powder? or solution? or device or inhaler))) adj4 (Cost or Costs or Budget* or Expens* or Expenditur* or Money* or financ* or Payment*)).ab,kw,ti.

8. "Costs and Cost Analysis"/

9. 6 and 8

10. 3 or 7 or 9

11. Controlled Clinical Trials as Topic/ or clinical studies as topic/ or Randomized Controlled Trials as Topic/ or clinical trials as topic/ or clinical trial? as Topic.mp. or randomised controlled trial? as Topic.mp. or research design/mt, st or Biomedical Research/mt, st

12. 10 and 11

13. limit 12 to yr="2015 -Current"

*Search strategy related to IMP services of hospital pharmacies*

Database: Ovid Medline(R) ALL

1. (hospital pharmacy or hospital pharmacies or pharmacy adj3 hospital? or pharmacies adj3 hospital? or pharmacy service? or pharmaceutical service? or pharmacy department?).ab,kw,ti
2. exp Pharmacy Service, Hospital/
3. 1 OR 2
4. Controlled Clinical Trials as Topic/ or clinical studies as topic/ or Randomized Controlled Trials as Topic/ or clinical trials as topic/ or clinical trial? as Topic.mp. or randomised controlled trial? as Topic.mp. or research design/mt, st or Biomedical Research/mt, st
5. (IMP or investigational medical product? or investigational medicinal product? or investigational drug or investigational drugs or trial medication? or trial drug?).ab,kw,ti.
6. exp drugs, investigational/
7. 4 OR 5 OR 6
8. 3 AND 7

Database: Ovid Embase

1. (hospital pharmacy or hospital pharmacies or pharmacy adj3 hospital? or pharmacies adj3 hospital? or pharmacy service? or pharmaceutical service? or pharmacy department?).ab,kw,ti
2. exp hospital pharmacy/
3. 1 OR 2
4. "controlled clinical trial (topic)"/ OR "randomized controlled trial (topic)"/ OR "clinical trial (topic)"/ OR clinical trial? as Topic.mp. or randomised controlled trial? as Topic.mp.
5. (IMP or investigational medical product? or investigational medicinal product? or investigational drug or investigational drugs or trial medication? or trial drug?).ab,kw,ti.
6. exp new drug/
7. 4 OR 5 OR 6
8. 3 AND 7
