# Supplementary material for: Investigational medicinal products, related costs and hospital pharmacy services for investigator-initiated trials: A mixed-methods study
Source: PLoS One. 2022 Mar 4;17(3):e0264427. doi: 10.1371/journal.pone.0264427 (PMC8896670; doi:10.1371/journal.pone.0264427)
Supplement: S2 Appendix — (DOCX) [file pone.0264427.s002.docx]

**Investigational Medicinal Products, related Costs and Hospital Pharmacy Services for Investigator-Initiated Trials: A mixed-methods study**

**S2 Appendix. PRISMA flow diagram**

*PRISMA flow diagram related to search for IMP costs*

Two case studies reported on cost items of clinical trials including IMPs and one survey examined costs of placebos in investigator-initiated clinical trials, but none of them compared planned and actual IMP costs; full-text articles excluded, with reasons
(n = 3)

Empirical studies on planned and actual costs of IMPs for clinical trials
(n = 0)

Records excluded
(n = 1659)

Full-text articles assessed for eligibility
(n = 3)

Records screened
(n = 1662)

Records after duplicates removed
(n = 1662)

Additional records identified through other sources
(n = 0)

Records identified through database searching

Medline: n= 521

EMBASE: n= 1411

*PRISMA flow diagram related to search for IMP services of hospital pharmacies*

Records excluded
(n =563)

Full-text articles assessed for eligibility
(n =70)

Records screened
(n =633)

Additional records identified through other sources
(n = 0)

Records identified through database searching

Medline: n= 224

EMBASE: n= 495

Records after duplicates removed
(n =633)

- Published before 2000 (n= 43)
- Legalisation on IMP preparation standard (n= 9)
- No abstract nor full text available (n=1)
- Full text in Japanese (n=1)
- Focus on pharmacist’ role (n=5)

Studies included in qualitative synthesis for the discussion section
(n = 11)
