# Supplementary material for: Investigational medicinal products, related costs and hospital pharmacy services for investigator-initiated trials: A mixed-methods study
Source: PLoS One. 2022 Mar 4;17(3):e0264427. doi: 10.1371/journal.pone.0264427 (PMC8896670; doi:10.1371/journal.pone.0264427)
Supplement: S4 Appendix — (DOCX) [file pone.0264427.s004.docx]

**Investigational Medicinal Products, related Costs and Hospital Pharmacy Services for Investigator-Initiated Trials: A mixed-methods study**

**S4 Appendix. Interview guide**

*Hospital Pharmacy Staff*

**1. Opening**

Thank hospital pharmacy staff for taking part in interview.

Explain conversation will be tape-recorded, transcribed and anonymized.

Tell them length of interview (15-20 minutes approx.), no interruptions / mobiles if possible.

Obtain info about previous experience in working with clinical researchers, trialists in particular.

**2. Regarding Services and Trial Investigators:**

- In your opinion, how do trial investigators know about the services of the Hospital Pharmacy regarding IMPs?
- How would you describe the level of knowledge trial investigators typically have about the provision of IMPs (including placebos) at hospital pharmacies?
- How do you inform trial investigators about IMP services for clinical trials (Website, 1-on-1 consultations, and presentations at certain events)?

**3. Regarding Costs:**

- In your opinion, what are the most frequent reasons for differences in planned and actual costs regarding IMPs in clinical trials?
- What are the difficulties you face, because of such differences?
- What are the difficulties you face when estimating (planned) costs for IMPs? What are the biggest uncertainties?
- From your expertise, how problematic is a difference in planned and actual costs (e.g. doubling of costs) for trial investigators?

**4. Regarding Process / System Level:**

- Do you think, that anything should be changed systematically/ on a system level in order to improve/ optimize the provision of IMPs for clinical trials?
- Anything that investigators should know/ consider before they come to you?
- What are your most important learned lessons from working with investigators regarding IMPs for clinical trials?

**5. Close of interview:**

Do you have any other comments/remarks you would like to make on the topic?

Thank pharmacists for their contribution, acknowledgement in final report.

Copy of results will be sent to them.

*Principal Investigators*

**1. Opening**

Thank PI for taking part in interview.

Explain conversation will be tape-recorded, transcribed and anonymized.

Tell them length of interview (15-20 minutes approx.), no interruptions / mobiles if possible

Obtain info about previous trial experience, position, and experience in working with hospital pharmacy.

**2. Background to PIs Experience**

- For how many years have you worked as a researcher with clinical trials?
- How many clinical trials have you conducted as PI/ Co-PI?

**3. Background to trial:**

- Who calculated the budget for your trial/ trials?
- Did you have help for the task (e.g. CTU)?
- If not:
- why not?

**4. Regarding IMPs**

- Did you consider different options regarding provision of IMPs for your trial (e.g. direct contact with pharmaceutical companies)? Or did you directly approach the hospital pharmacy?
- Why did you decide to go to the hospital pharmacy to manufacture the IMPs?
- How did you know that the hospital pharmacy offers this service?
- Do you know which services are offered by the hospital pharmacy to support trial investigators (e.g. destruction of remaining IMPs, archiving records, preparing & performing randomization, blinding, etc.)?

**5. Regarding Placebo**

- Did you need a placebo in your trial?

**If Yes:**

- What kind of placebo was used?

**If matching:**

- In which ways was the placebo matching?
- Did it fulfil your expectations?

**6. Regarding IMP Provision and Costs:**

- The costs for the IMP provision in your trial differed substantially from the planned costs.

What were the reasons for this?

Was this a problem for you/your research team?

- OR: The costs for the IMP provision in your trial matched well with the planned costs.

What were the important points to get this right?

What do you think are the most frequent reasons if planned and actual costs do not match?

- When writing the trial protocol or when you first read/ familiarized yourself with the protocol, did you **anticipate potential problems with IMP provision?** Which problems did you encounter?

**7. Regarding Process / System Level:**

- In general, do you think that anything should be changed systematically/on a system level in order to improve/ optimize the provision of IMPs for clinical trials?
- Anything that could/ should be changed at the Hospital Pharmacy to improve the process?
- What are your most important learned lessons from working with the hospital pharmacy?

**8. Close of interview:**

Do you have any other comments/remarks you would like to make on the topic?

Thank trialist for his/her contribution, acknowledgement in final report.

Copy of results will be sent to them.

*Clinical Trial Unit Staff:*

**1. Opening**

Thank Clinical Trial Unit staff for taking part in interview.

Explain conversation will be tape-recorded, transcribed and anonymized.

Tell them length of interview (15-20 minutes approx.) no interruptions / mobiles if possible.

Obtain info about previous experience in working with clinical researchers, trialists in particular and with hospital pharmacy.

**2. Regarding Services and Trial Investigators:**

- In your opinion, how do trial investigators know about the services of the Hospital Pharmacy regarding IMPs?
- How would you describe the level of knowledge trial investigators typically have about the provision of IMPs (including placebos) at hospital pharmacies?
- How does the hospital pharmacy inform trial investigators about IMP services for clinical trials (Website, 1-on-1 consultations, and presentations at certain events)?

**3. Regarding Costs:**

- In your opinion, what are the most frequent reasons for differences in planned and actual costs regarding IMPs in clinical trials?
- What are the difficulties you face when estimating (planned) costs for IMPs? What are the biggest uncertainties?
- From your expertise, how problematic is a difference in planned and actual costs (e.g. doubling of costs) for trial investigators?

**4. Regarding Process / System Level:**

- Do you think that, anything should be changed systematically/ on a system level in order to improve/optimize the provision of IMPs for clinical trials?
- Anything that investigators should know/ consider when working with hospital pharmacies?
- What are your most important learned lessons from working with investigators regarding IMPs for clinical trials?

**5. Close of interview:**

Do you have any other comments/remarks you would like to make on the topic?

Thank the person for their contribution, acknowledgement in final report.

Copy of results will be sent to them.
