# Supplementary material for: Investigational medicinal products, related costs and hospital pharmacy services for investigator-initiated trials: A mixed-methods study
Source: PLoS One. 2022 Mar 4;17(3):e0264427. doi: 10.1371/journal.pone.0264427 (PMC8896670; doi:10.1371/journal.pone.0264427)

**Investigational Medicinal Products, related Costs and Hospital Pharmacy Services for Investigator-Initiated Trials: A mixed-methods study**

**S5 Appendix. Figure on relative increase in planned vs. actual costs of IMPs**


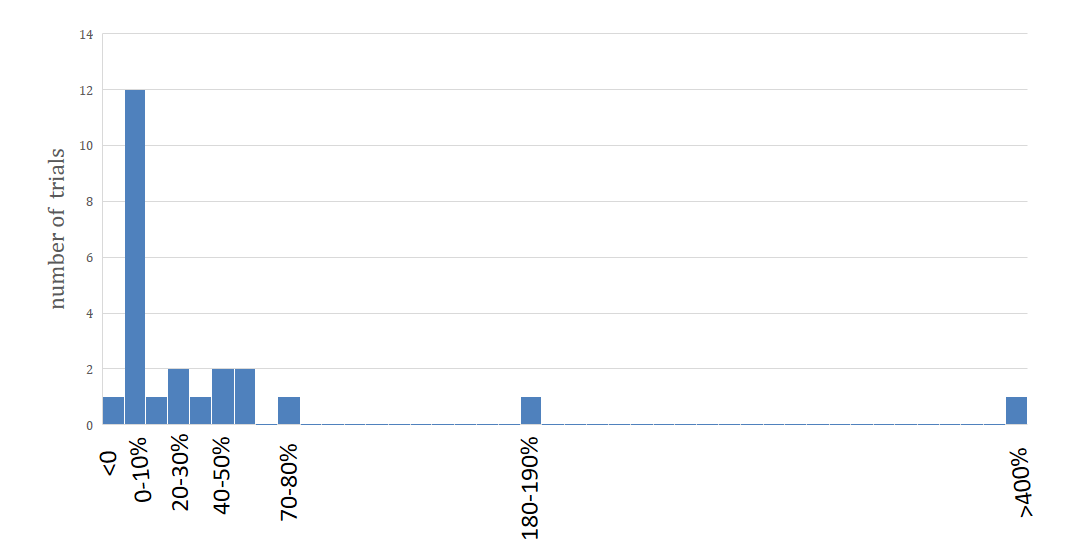

Supplement: S5 Appendix — (DOCX) [file pone.0264427.s005.docx]
