# Supplementary material for: Investigational medicinal products, related costs and hospital pharmacy services for investigator-initiated trials: A mixed-methods study
Source: PLoS One. 2022 Mar 4;17(3):e0264427. doi: 10.1371/journal.pone.0264427 (PMC8896670; doi:10.1371/journal.pone.0264427)
Supplement: Attachment — Submitted filename: Response to Reviewers.docx [file pone.0264427.s006.docx]

To

Ismaeel Yunusa, PharmD, PhD

Academic Editor
PLOS ONE

Basel, February 4, 22

Ref. ATH

**MS ID#:** PONE-D-21-09088

**First revision:** Investigational medicinal products, related costs and hospital pharmacy services for investigator-initiated trials: A mixed-methods study

Dear Dr. Yunusa,

We thank you for reviewing our manuscript and for considering a revised version for publication in *PloS One*. We have addressed all of the points raised by you and the reviewers and we are grateful for the constructive comments that helped to further improve our manuscript.

Please consider the following **Editor’s specific comments (No. 2 and 3)** in order to change in online form on our behalf.

**The authors received no specific funding for this project. VRM is supported by the Swiss National Science Foundation through an Early Postdoc.Mobility career grant (P2BEP3_191798).**

- **The authors have declared that no competing interests exist.**

All contributors have approved this revised version of the manuscript and fulfill criteria for authorship.

We hope that the revised manuscript meets your and the reviewers’ expectations and that it can be accepted for publication in the present form.

Sincerely yours,

Ala Taji Heravi

Basel Institute for Clinical Epidemiology and Biostatistics, Department of Clinical Research

University Hospital Basel, Basel, Switzerland; Email: [ala.tajiheravi@usb.ch](mailto:ala.tajiheravi@usb.ch)

**Point by point reply:**

MS ID#: PONE-D-21-09088

**First revision:** Investigational medicinal products, related costs and hospital pharmacy services for investigator-initiated trials: A mixed-methods study

The editor’s and reviewers’ comments are in **bold** font and our replies in regular font.

A) **Editor’s specific comments**

**1. Please ensure that your manuscript meets PLOS ONE's style requirements, including those for file naming. The PLOS ONE style templates can be found at** 
[**https://journals.plos.org/plosone/s/file?id=wjVg/PLOSOne_formatting_sample_main_body.pdf**](https://journals.plos.org/plosone/s/file?id=wjVg/PLOSOne_formatting_sample_main_body.pdf) **and** 
[**https://journals.plos.org/plosone/s/file?id=ba62/PLOSOne_formatting_sample_title_authors_affiliations.pdf**](https://journals.plos.org/plosone/s/file?id=ba62/PLOSOne_formatting_sample_title_authors_affiliations.pdf)

**Author reply:** Please find our changes in the revised manuscript.

**2. Thank you for stating the following financial disclosure: "There were no specific funds available for this project."
At this time, please address the following queries:**

**a) Please clarify the sources of funding (financial or material support) for your study. List the grants or organizations that supported your study, including funding received from your institution. 
b) State what role the funders took in the study. If the funders had no role in your study, please state: “The funders had no role in study design, data collection and analysis, decision to publish, or preparation of the manuscript.”
c) If any authors received a salary from any of your funders, please state which authors and which funders.
d) If you did not receive any funding for this study, please state: “The authors received no specific funding for this work.”**

**Please include your amended statements within your cover letter; we will change the online submission form on your behalf.**

**Author reply:** The amended statement is mentioned in the cover letter as requested.

**3. Thank you for stating the following in your Competing Interests section: "All authors declare no financial relationships with any organization that might have an interest in the submitted work and no other relationships or activities that could appear to have influenced the submitted work."**

**Please complete your Competing Interests on the online submission form to state any Competing Interests. If you have no competing interests, please state "The authors have declared that no competing interests exist.", as detailed online in our guide for authors at** [**http://journals.plos.org/plosone/s/submit-now**](http://journals.plos.org/plosone/s/submit-now) 
 **This information should be included in your cover letter; we will change the online submission form on your behalf.**

**Author reply:** The amended statement is mentioned in the cover letter as requested.

**4. Your ethics statement should only appear in the Methods section of your manuscript. If your ethics statement is written in any section besides the Methods, please move it to the Methods section and delete it from any other section. Please ensure that your ethics statement is included in your manuscript, as the ethics statement entered into the online submission form will not be published alongside your manuscript.**

**Author reply:** We moved the ethics statement to the Methods section as requested.

**5. Please upload a copy of Supporting Information S1-S5 Appendix which you refer to in your text on page 34.**

**Author reply:** We had already uploaded a copy of the Supporting Information as a zip file in March 2021 with the original submission. We have now uploaded Supplementary word files with sections S1-S5 with our revised manuscript.

B) **Reviewers' comments:**

**Reviewer #1:**

**This paper is an interesting paper and it brings to light challenges experienced in conducting clinical trials. Here are some of the comments for the authors to take note of:
1. Last paragraph in the Quantitative results, where the authors discussed on the reason for cost differences, I would suggest they present it in tabular form for easier reading as it passes an important information.**

**Author reply:** We thank the reviewer for this suggestion, which we followed. We added a corresponding table (new Table 3) to the revised manuscript.

**2. In the discussion, the summary of findings seems quite long and was a repetition of what was already mentioned in the Results section. I would suggest the authors discuss their results together with the comparison with other studies (although the mentioned few studies available for comparison) to prevent multiple repetitions.**

**Author reply:** We see the reviewer’s point and substantially shortened the “summary of findings” section of the Discussion in order to minimize repetitions.

**3. Line 292 replace higher than ‘then’ to higher than ‘the’**

**Author reply:** We apologize for this typo. It is corrected now.

**Reviewer #2:**

**Overall, this is an interesting and well written manuscript that picks up on a completely unstudied area that deserves further investigation. More details should be provided in the background section. The qualitative part of the study is quite strong. I have some observations to the quantitative part of the study. I also wonder this is in fact a mixed methods design. There is a qualitative and a quantitative part, while I wonder whether the connection between them is strong or whether both parts could had also been published as separate parts.

I have the following comments:
1. consider whether the title is appropriate. What would be a non-empirical mixed-methods-study? Aren´t they all empirical?**

**Author reply:** Thank you for this suggestion. We changed the title to the following:

*“Investigational medicinal products, related costs and hospital pharmacy services for investigator-initiated trials: a mixed-methods study”*

**2. as the cut-off of 10% is crucial for your analysis its definition must be placed in the abstract**

**Author reply:** Thank you for this suggestion. We added the following sentence to the Methods section of the abstract:

*“Our working definition for a substantial cost difference was that the actual IMP costs were more than 10% higher than the planned IMP costs in a trial.”*

We also added this definition to each table legend in which we stratified included trials by substantial cost differences (with vs without cost difference of 10%).

**3. The introduction would benefit from more background information as this topic will be rather new for most readers. - With IMP you focus on one potential contributor to the costs. What are the other contributors?**

**Author reply:** Thank you for raising this point. We agree and added the following information to the second paragraph of the Introduction section:

*“Various tasks are necessary for the successful conduct of an IIT, all of them generating costs. While some costs are covered through collaborators with regular positions in the hospital, specific trial related costs require third party funding. This can be, but is not limited to salary costs of study staff, fees of ethical or other regulatory authorities, database set-up, data monitoring, laboratory costs, insurance costs etc. They all sum up to the overall costs of a trial [**12**,* *13**]. There is little empirical evidence on overall cost structures and individual cost components [**12**,* *13**].”*

**4. line 63: 'In IICTs testing drug substances' ... I am not sure what this does mean? Does this refer to pharmacological trials (drug substance is at least part of an intervention)? For my understanding, IMP costs can also occur in a surgical trial. Please clarify your concepts and definitions. In particular clearly define IMP.**

**Author reply:** Thank you for the opportunity to better clarify this issue. For our study we used a working definition for an ‘Investigational medicinal product (IMP)’ based on the EU Clinical Trial Regulation (<https://eur-lex.europa.eu/legal-content/EN/TXT/PDF/?uri=CELEX:32014R0536&from=EN%20> ) which means a medicinal product that is being tested or used at least as part of an intervention or used as a reference, including a placebo, in a trial.

We agree with the reviewer that IMP costs can also occur e.g. in a surgical trial. Therefore, we specified “at least as part of an intervention…”.

To clarify this better we re-phrased the beginning of the corresponding sentence as follows:

*“In IITs in which an active substance or a placebo is being tested at least as part of an intervention or used as a reference…”*

Moreover, we added our IMP definition as a first para in the Methods section of the revised manuscript as follows:

*“We chose a working definition of an IMP for our study according to the EU Clinical Trial Regulation referring to a medicinal product that is being tested or used at least as part of an intervention or as a reference (including placebo) in a trial* [16]*. IMP costs included any of the following: Purchasing price, repacking, labelling, development and manufacturing of one or several batches of IMP, contributing to regulatory approval, storage, logistics, randomisation, and emergency unblinding, if needed.”*

**5. Following this, I fail to understand whether your included trials tested drug substances?**

**Author reply:** Thank you for the opportunity to clarify this. We included IITs in our study, for which the local hospital pharmacy provided services with respect to IMPs. Regarding the definition of an IMP and our answer to the previous question 4, this means that the substance was tested as (part of) an intervention or was the reference substance used in a trial.

**6. line 79: does the time span relate to the duration of the trials or to receiving the invoice?**

**Author reply:** Thank you for your comment. We are referring here to the time interval of pharmacy invoices. To clarify this better for readers we re-phrased the corresponding section as follows:

*“All IITs (randomized and non-randomized) that used any service regarding IMPs at the hospital pharmacy of the University Hospital Basel where the hospital pharmacy invoiced its services between January 2014 and June 2020 were included; before 2014 the administrative system of the hospital pharmacy had been re-organized several times.”*

**7. I have no idea what is meant by hospital pharmacy documents. Please explain more in detail. It is important for the reader to gain sufficient knowledge of the data source. I think that this information can be easily provided in the supplement.**

**Author reply:** Thank you for this comment. To make it clearer for readers what material we used for data extraction, we re-phrased the corresponding sentence as follows:

*“We used administrative trial files at the hospital pharmacy that contained (among other documents) trial protocols, descriptions of provided services regarding IMPs, and issued invoices to extract the following information:…”*

**8. line 117: add 'were'**

**Author reply:** We thank the reviewer for spotting this typo, which we corrected.

**9. why did you only extract whether there was 'Swiss National Science Foundation support'? What about other sources of funding? There might also be internal hospital funding, for example.**

**Author reply:** Thank you for providing us with an opportunity to clarify this point. Our intention was to see whether IITs that successfully completed a competitive selection process of a national funding agency would perform differently in terms of discrepancies between planned and actual IMP costs. We rephrased the corresponding item in Table 1 as follows:

*“Competitive funding from the Swiss National Science Foundation”*

**10. was there a protocol for your study? please state. This needs to be state as the cut-off of 10% additional costs looks a bit arbitrary to me. From my perspective, 10% is not much and is something that can be expected. Is there any rationale for the 10%? Otherwise you should consider whether a sensitivity analysis might be useful. It definitely needs to be discussed as you had a special focus on principal investigators where the trial had a substantial difference. If the cut-off would have been set higher (eg. 20%) you might not have been able to recruit investigators for your interviews. Please provide more details on the actual absolute difference in costs (median, IQR, range).**

**Author reply:** Thank you for providing us with an opportunity to clarify several points.

This manuscript is part of the MSc thesis of Ms. Taji Heravi. A protocol was written and submitted to the committee of the Science faculty in Basel, however, it was not published in any way.

We agree with the reviewer that the cut-off of 10% is somewhat arbitrary, but we discussed it with several clinical researchers and CTU staff members beforehand and agreed that financial resources for IITs are always scarce and precious and therefore settled on these 10%. If one considers the histogram in S5 Appendix of the supplementary material, it is clear that a higher cut-off of e.g. 20% would not change our results, because only 1 trial would switch groups from substantial difference to no substantial difference.

To better clarify this in the revised manuscript, we added the following sentence to the Data Analysis paragraph in the Methods section:

*“The chosen cut-off was based on deliberations with involved stakeholders stressing that financial resources for IITs are always scarce and precious.”*

Moreover, we added the following to the limitations in the Discussion section:

*“Second, our chosen cut-off of more than 10% difference between planned an actual IMP costs to be considered substantial could be perceived as arbitrary and too low rendering our results little robust. However, as shown in S5 Appendix a higher cut-off of e.g. 20% would be unlikely to change our results since only one trial would switch categories from substantial difference to no substantial difference.”*

We now provide more details on absolute cost data in terms of median, IQR, and range in Table 1.

**11. I wonder whether the results in table 2 can be described in a more informative way. You have data on IMP-verum nested in trials. Your n is quite los, so I am not sure that this can be considered at all. However, as a minimal solution I would suggest to describe in how many studies more than one IMP-varum was used.**

**Author reply:** Thank you for this comment. We followed your suggestion and added the following information to the Table legend:

*“* There were 5 trials with more than one IMP-verum, 3 trials with more than one IMP-placebo and 2 trials without any IMP-placebo per trial.”*

**12. the qualitative part of the study is well described and informative; the same is true for the discussion.**

**Author reply:** We thank the reviewer for the positive feedback.

**Reviewer #3:**

**Thank you for giving me the opportunity to review this interesting manuscript. I have only some minor suggestions for improvement.
1. Can you give more detail what the costs of IMP include and how these were determined? Are these only the purchasing prices? As the pharmacy service was also involved in preparing (e.g. packing) the IMP this would that personal costs and thus a large share of costs were not considered.**

**Author reply:**

The costs of IMPs refer not only to the purchasing price, but also to the pharmacy related services. These services differed with respect to the trial design and duration, and usually included repacking, labelling, development and manufacturing of one or several batches of IMP, contributing to regulatory approval, storage, logistics, randomisation, and emergency unblinding, if needed.

To better clarify this for readers we added the following to the first para of the Methods section:

*“IMP costs included any of the following: Purchasing price, repacking, labelling, development and manufacturing of one or several batches of IMP, contributing to regulatory approval, storage, logistics, randomisation, and emergency unblinding, if needed.”*

**2. I would suggest to report the average cost per patient instead of absolute costs to improve comparability and interpretation in general.**

**Author reply:** Thank you for your suggestion. We believe that absolute costs and costs per patient are both associated with advantages and disadvantages. Therefore, we added further lines to Table 1 and now additionally provide average IMP costs per patient for both planned and actual IMP costs.

**3. Are the reported cost the total cost in a certain trial?**

**Author reply:** Thank you for the opportunity to clarify this point. The reported costs were the total costs with respect to IMP services in a certain trial.

**4. Were data on costs missing? If so, it would be good to have more information on missing cost data, considering that if principle investigators guessed this it is probably imprecise.**

**Author reply:** Thank you for your question. Cost data on IMP services were complete for all included trials (no missing data). However, the “IMP fraction of total trial costs in %” presented in Table 1 was estimated by the principal investigator in three trials and not provided in five trials. We added the following information to the legend of Table 1:

*“** Based on available data on overall trial costs; for three trials overall costs were not available and principal investigators then estimated the IMP fraction of total trial costs* *, and for five trials principal investigators did respond to provide us with IMP fraction of total trial costs.”*
